# Associations of toll-like receptor polymorphisms with systemic lupus erythematosus: A meta-analysis

**DOI:** 10.1016/j.heliyon.2024.e27987

**Published:** 2024-03-12

**Authors:** Young Ho Lee, Gwan Gyu Song

**Affiliations:** Department of Rheumatology, Korea University Medicine, Korea University College of Medicine, Seoul, South Korea

**Keywords:** Toll-like receptor 7, Toll-like receptor 4, Polymorphism, Systemic lupus erythematosus, Meta-analysis

## Abstract

**Objective:**

The objective of this study was to examine whether polymorphisms in toll-like receptors 7 and 4 (TLR7 and 4) contribute to vulnerability to systemic lupus erythematosus (SLE).

**Methods:**

We searched MEDLINE, Embase, and Web of Science for relevant articles and performed a meta-analysis to investigate the relationship between TLR7 rs179008, rs3853839, rs1790010, TLR4 rs4986791, and rs798690 polymorphisms and SLE.

**Results:**

Eighteen studies and 16 papers including 8022 patients with SLE and 9822 healthy controls were retrieved. Meta-analysis revealed that the TLR7 rs179008 T variant was not associated with SLE (OR = 1.008, 95% CI = 0.849–1.394, P = 0.504). Ethnic classification revealed no association between the TLR7 rs179008 T gene and SLE in either European or Latin American groups. Additionally, homozygous comparison, recessive, and dominant models revealed no association between the TLR7 rs179008 variant and SLE. In contrast, a significant association between SLE and the TLR7 rsrs3853839 GG + GA allele (OR = 2.135, 95% CI = 1.502–3.035, <0.001; OR = 23.20, 95% CI = 14.13–38.08, <0.001) was observed in the Arab and Asian groups. The T variant of TLR7 rsrs179010 was also associated with SLE in Asians (OR = 1.177, 95% CI = 1.048–1.321, P = 0.006). In contrast, the TLR4 rs4986791 variant was not associated with SLE in Europeans when allele, homozygous comparison, recessive, and dominant models were used. Furthermore, no association between the TLR4 rs4986790 variant and SLE risk in Europeans was found using any genomic model.

**Conclusions:**

Meta-analysis revealed that the TLR7 rs3853839 variant is associated with SLE risk in Asians and Arabs and that TLR7 rs179010 is associated with SLE in Asians. However, TLR7 rs179008, TLR4 rs4986791, and TLR rs798690 polymorphisms were not associated with SLE risk.

## 1 Introduction

Systemic lupus erythematosus (SLE) is a complex inflammatory condition that can potentially damage various organ systems, including the skin, joints, and kidneys. SLE has a substantial hereditary component, and many candidate genes have been investigated as possible risk factors [1,2]. Toll-like receptors (TLRs) have been shown to play a major role in the etiology of SLE by identifying and reacting with endogenous and external antigens [3]. TLRs function as the pattern-recognition receptors of the natural immune system, and are crucial components that trigger inflammatory reactions in response to pathogens and signs of risk from the host [4].

Recent genetic linkage research suggests that TLR7 and TLR4 single nuclear polymorphisms (SNPs) may increase vulnerability to SLE. Both RNA and lipopolysaccharide recognition and the initiation of subsequent signaling pathways depend on these TLRs [5]. Although the findings of these studies are unclear, it has been proposed that TLR7 rs179008, rs3853839, and rs1790010 polymorphisms may be risk factors for SLE. Similar studies have been conducted on TLR4 rs4986791 and rs798690 polymorphisms associated with SLE, with conflicting results [[6], [7], [8], [9], [10], [11], [12], [13], [14], [15], [16], [17], [18], [19], [20], [21]].

Considering the abundance of literature on the association between TLR variants and SLE, a methodical and rigorous strategy is necessary to compile available data. A meta-analysis is a mathematical method wherein data from various studies are combined, and enables a more accurate assessment of the relationship between a genetic variation and disease risk [22,23]. This method can facilitate the discovery of patterns and causes of variability that may not be apparent in individual studies [[24], [25], [26]]. In this study, we performed a meta-analysis of the literature to examine the relationship between TLR7 and TLR4 variants and SLE to identify possible causes of study variability and provide a more reliable assessment of the relationship between these genetic variations and SLE risk. We focused on TLR4 rs4986791 and rs798690 SNPs and TLR7 rs179008, rs3853839, and rs1790010 variants, which have received the most attention for their association with SLE.

## 2 Materials and methods

### 2.1 Locating relevant studies and assembling information

We searched the literature to determine the association between TLR7 variants and SLE. We used MEDLINE, Embase, and Web of Science to identify papers examining TLR7 or TLR4 genetic variants in SLE cases (from inception to March 2023). We searched through the reference lists of the selected papers to find additional research not listed in the databases for combinations of phrases such as “toll-like receptor 7,” “toll-like receptor 4,” “polymorphism,” and “lupus.” No restrictions based on geography, language, sex, or ethnicity were set. Only studies that met the following requirements were considered: (1) they had to have been case-control studies; (2) they had to have examined TLR7 SNPs in SLE and control groups; and (3) they had to have sufficient information to compute an odds ratio (OR). Studies with identical data and those in which the number of null and wild-type alleles could not be determined were excluded. Data from the original studies were collected for the meta-analysis by two assessors who were unaware of the primary study findings. A third critic was enlisted when disputes arose between the evaluators. We collected data on each study author, publication year, study demographics, nationality of the community under investigation, and case-control statistics, and the Newcastle–Ottawa measure was used to assess the studies [27]. The approach was considered to have good fidelity if the result was between six and nine.

### 2.2 Assessments of data relationships

A chi-square test was performed to determine whether the stated genotype frequency was in Hardy–Weinberg equilibrium (HWE). Meta-analyses were performed using four types of genetic models: (1) allelic contrast, (2) homozygous contrast, (3) recessive, and (4) dominant. Additionally, subgroup studies based on ethnicity were conducted to investigate differences among ethnic groups, and collective rates (ORs) and 95% confidence intervals (CIs) were estimated. Cochran's Q test was used to evaluate the variability and variance both within and between trials. The results of all the examined studies served as the foundation for this measurement of variability. The I^2^ value, which varies from 0% to 100% and denotes the percentage of variation between studies that could be ascribed to variables other than chance [28], was used to assess the impact of heterogeneity. I^2^ levels between 25% and 50% were considered low, 50–75% were considered intermediate, and >75% were considered high. Researchers who use the fixed-effects approach assume that the influence of a specific hereditary factor on disease risk remains constant across studies, and that any variations are the result of arbitrary chance. When estimating real influence, the random-effects model considers both selection errors within a study and variations between studies. Both models yielded similar findings when the study groups were comparable. However, when they were not, the results of the random-effects model generally had larger CIs than those of the fixed-effects model. A random-effects model was used when substantial between-study variability was present (heterogeneity p-value 0.1 or I^2^ > 50%) [29]. Statistical analyses were performed using Comprehensive Meta-Analysis software (Biostat Inc., Englewood, NJ, United States).

### 2.3 Analyzing the degree of bias in publication bias

Although funnel models could have been used to identify publication bias, funnel plotting requires numerous studies of various sizes and involves biased assessment. Therefore, Egger's linear regression test [30], which evaluates funnel plot inequality using a natural logarithmic measure of ORs, was used to evaluate publication bias.

## 3 Results

### 3.1 Pooled research

**Fig. 1:**
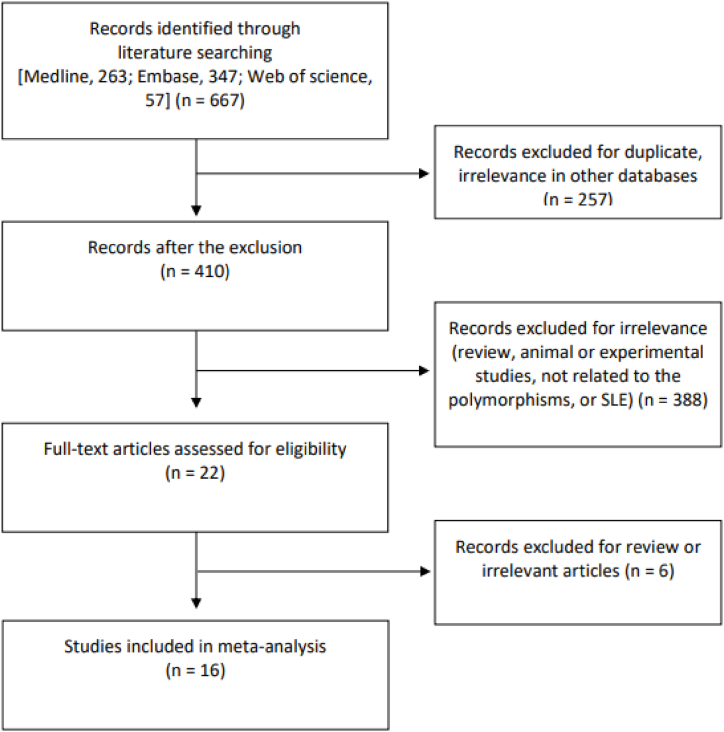
Procedure for selection of articles to include in the meta-analysis.

A total of 667 articles were identified using both computerized and manual search strategies, of which 22 met the criteria for full-text evaluation. Six of these articles were excluded as they lacked polymorphism data; therefore, the inclusion criteria were met by a total of 16 articles [[6], [7], [8], [9], [10], [11], [12], [13], [14], [15], [16], [17], [18], [19], [20], [21]]. Two studies that met these criteria included data from two distinct groups receiving individual care [7,17]. The meta-analyses of the final 18 studies therefore included 8022 patients with SLE and 9822 controls (Table 1, Fig. 1). The meta-analyses focused on the following five SNPs: TLR7 rs179008, rs3853839, rs1790010, TLR4 rs4986791, and rs798690. All the included studies were rated between 6 and 8 on a quality-grading scale, indicating high quality. The main findings of studies that linked TLR7 and 4 polymorphisms to diseases are summarized in Table 1.

**Table 1:** Characteristics of the studies included in the meta-analysis.

Author (Ref)	Country	Ethnicity	Subjects		Polymorphisms studied
			Case	Control	
Azab, 2022 [6]	Egypt	Arab	100	100	TLR7 rs3853839
Pacheo-1, 2022 [7]	Mexico	Latin American	90	102	TLR7 rs179008
Pacheo-2, 2022 [7]	Mexico	Latin American	151	121	TLR7 rs179008
Elloumi, 2022 [8]	Tunisia	Arab	100	201	TLR4 rs4986790, rs4986791
Aranda-Uribe, 2021 [9]	Mexico	Latin American	283	424	TLR4 rs4986790, rs4986791
Elloumi, 2021 [10]	Tunisia	Arab	93	170	TLR7 rs3853839
Bashir, 2021 [11]	Parkistan	Asian	80	80	TLR7 rs179008
Raafat, 2018 [12]	Egypt	Arab	50	50	TLR7 rs3853839
Rupasree, 2015 [15]	India	Asian	194	223	TLR4 rs4986790, rs4986791
Enevold, 2014 [16]	Denmark	European	132	412	TLR7 rs3853839, rs179010
Wang, 2014 [13]	Taiwan	Asian	795	1162	TLR7 rs3853839, rs179010
Bogaczewicz, 2013 [14]	Poland	European	60	100	TLR4 rs4986791
Santos-1, 2012 [17]	Brazil	European	259	114	TLR7 rs179008
Santos-1, 2012 [17]	Brazil	African	83	43	TLR7 rs179008
Kawasaki, 2011 [18]	Japan	Asian	344	274	TLR7 rs3853839, rs179010
Shen, 2010 [20]	China	Asian	4334	4940	TLR7 rs3853839
Sanchez, 2009 [19]	Spain	European	752	1107	TLR7 rs179008
Sanchez, 2004 [21]	Spain	European	122	199	TLR4 rs4986790, rs4986791

Ref: reference, *TLR7, 4: Toll-like receptor 7, 4*.

Meta-analysis of the association between TLR7 rs179008, rs3853839, and rs1790010 polymorphisms and SLE.

**Fig. 2:**
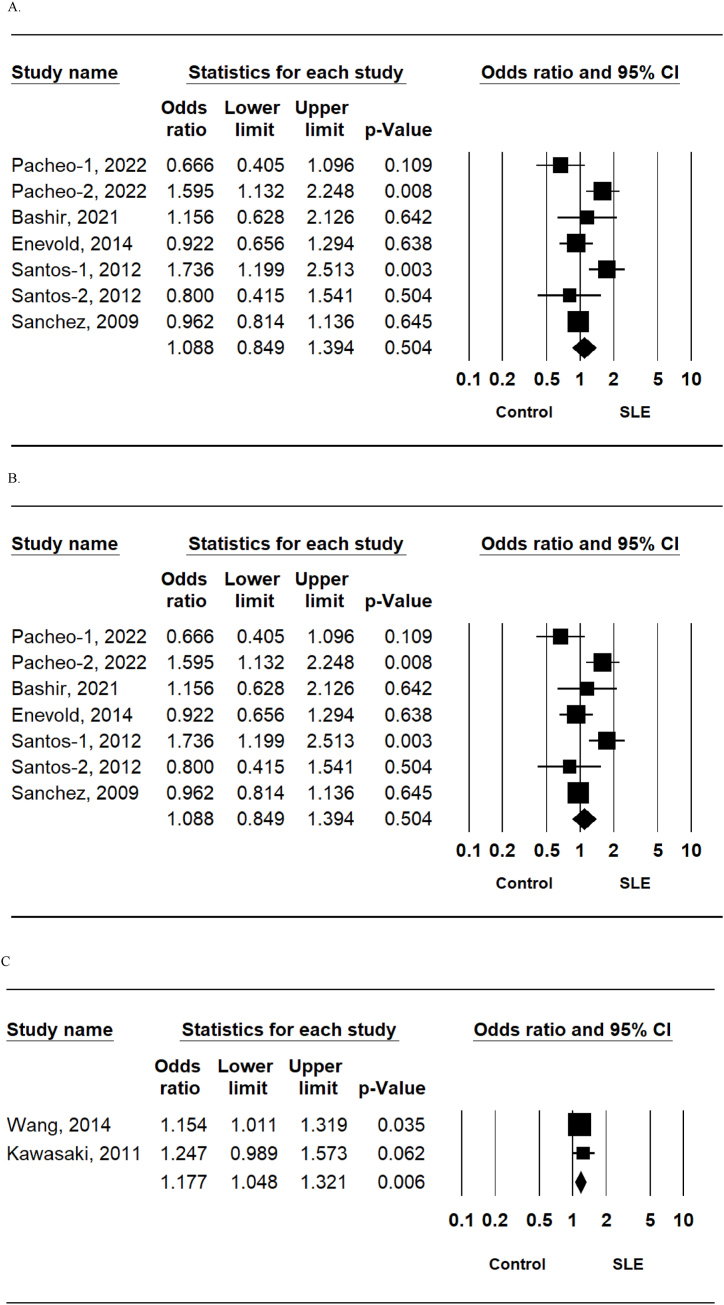
Meta-analysis of the genetic association between systemic lupus erythematosus susceptibility and the toll-like receptor 7 rs179008 T allele (a), rs3853839 GG + GC genotype (b), and rs179010 T allele (c) polymorphisms.

The meta-analysis revealed no association between SLE and the TLR7 rs179008 T allele (OR = 1.008, 95% CI = 0.849–1.394, P = 0.504) (Table 2, Fig. 2A). Additionally, ethnic stratification revealed no association between the TLR7 rs179008 T allele and SLE in either European or Latin American populations (Table 2). Furthermore, the homozygous contrast, recessive, and dominant models revealed no association between TLR7 rs179008 polymorphism and SLE (Table 2). In contrast, the meta-analysis revealed a significant association between SLE and the TLR7 rsrs3853839 GG + GA genotype in the overall population (OR = 3.479, 95% CI = 1.231–9.836, P = 0.038) as well as in Arab and Asian populations (OR = 2.135, 95% CI = 1.502–3.035, <0.001; OR = 23.20, 95% CI = 14.13–38.08, <0.001) (Table 2, Fig. 2B). Additionally, the TLR7 rsrs179010 T allele was associated with SLE in Asians (OR = 1.177, 95% CI = 1.048–1.321, P = 0.006) (Table 2, Fig. 2C).

**Table 2:** Meta-analysis of the association between the TLR7 rs179008, rs3853839 and rs179010 polymorphisms and SLE.

Polymorphism	Population	No. of studies	Test of association			Test of heterogeneity		
			OR	95% CI	*P*-value	Model	*P*-value	I^2^
Rs179008 T vs. A allele	Overall	7	1.008	0.849–1.394	0.504	R	0.0005	67.3
	European	3	1.128	0.798–1.594	0.495	R	0.013	76.9
	Latin American	2	1.051	0.447–2.472	0.909	R	0.005	87.5
TT vs. TA + AA (Recessive)	Overall	6	1.243	0.769–2.007	0.374	F	0.246	25.1
	European	2	1.764	0.886–3.513	1.106	F	0.237	28.6
	Latin American	2	0.630	0.074–5.346	0.672	R	0.091	64.9
TT + TA vs. AA (Dominant)	Overall	6	1.363	0.769–2.415	0.289	R	<0.001	81.2
	European	2	1.220	0.617–2.414	0.567	R	0.022	80.9
	Latin American	2	2.504	0.190–32.99	0.485	R	<0.001	95.0
TT vs. AA	Overall	6	1.418	0.592–3.396	0.433	R	0.014	64.8
	European	2	1.827	0.911–3.666	0.090	R	0.146	52.5
	Latin American	2	1.481	0.022–99.37	0.855	R	0.002	89.5
Rs3853839 G vs. C allele	Overall	6	1.228	0.942–1.602	0.130	R	<0.001	89.1
	Arab	2	1.394	0.874–2.224	0.163	R	0.122	68.2
	Asian	3	1.074	0.727–1.588	0.719	R	<0.001	95.0
GG vs. GC + CC (Recessive)	Overall	4	1.519	1.165–1.980	0.002	F	0.412	0
	Arab	2	1.756	0.990–3.118	0.054	F	0.611	0
	Asian	1	1.340	0.974–1.844	0.072	NA	NA	NA
GG + GC vs. CC (Dominant)	Overall	5	3.479	1.231–9.836	0.019	R	<0.001	94.6
	Arab	3	2.135	1.502–3.035	<0.001	F	0.576	0
	Asian	1	23.20	14.13–38.08	<0.001	NA	NA	N0A
GG vs. CC	Overall	4	2.030	1.380–2.984	<0.001	F	0.541	0
	Arab	2	1.649	0.879–3.091	0.119	F	0.341	0
	Asian	1	2.026	1.125–3.649	0.019	NA	NA	NA
Rs179010 T vs. C allele	Asian	2	1.177	1.048–1.321	0.006	F	0.572	0

F: Fixed effect model; R: Random effect model, NA: Not available.

### 3.2 Meta-analysis of TLR4 rs4986791 and rs798690 polymorphisms and SLE

**Fig. 3:**
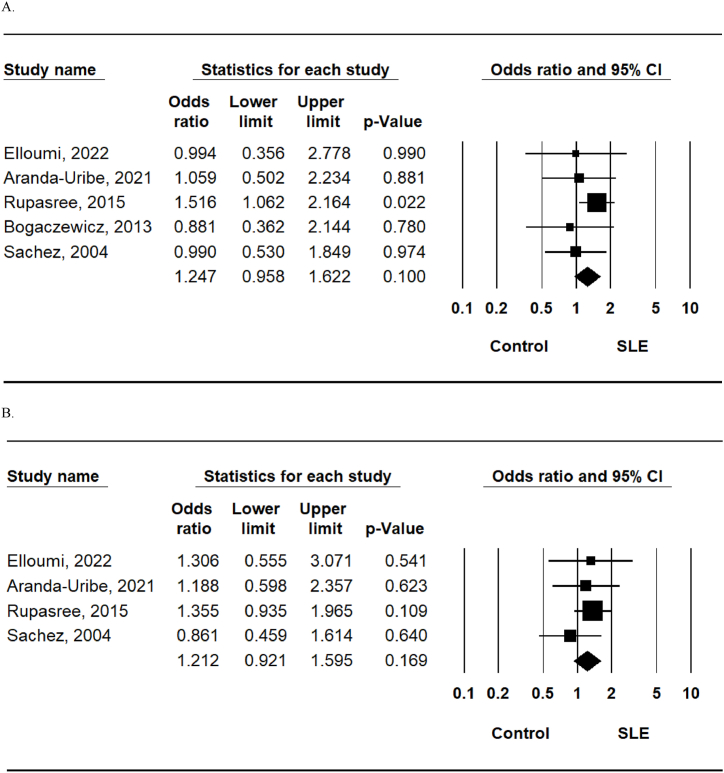
Meta-analysis of the allelic association between systemic lupus erythematosus susceptibility and the toll-like receptor 4 polymorphisms at rs4986791 (a) and rs4986790 (b).

Allele, homozygote comparison, recessive, and dominant models revealed no association between SLE and the TLR4 rs4986791 polymorphism in Europeans (Table 3, Fig. 3A). Additionally, no genetic models revealed an association between TLR4 rs4986790 polymorphism and SLE risk in Europeans (Table 3, Fig. 3B).

**Table 3:** Meta-analysis of the association between the TLR4 rs4986791 and rs4986790 polymorphisms and SLE.

Polymorphism	Population	No. of studies	Test of association			Test of heterogeneity		
			OR	95% CI	*P*-value	Model	*P*-value	I^2^
Rs4986791 T vs. C allele	Overall	5	1.247	0.958–1.622	0.100	F	0.620	0
	European	2	0.952	0.571–1.588	0.851	F	0.834	0
	Asian	1	1.516	1.062–2.164	0.022	NA	NA	NA
TT vs. TC + CC (Recessive)	Overall	3	2.230	0.636–7.815	0.210	F	0.337	8.13
	European	1	0.322	0.015–6.773	0.466	NA	NA	NA
	Asian	1	4.136	0.849–20.15	0.079	NA	NA	NA
TT + TC vs. CC (Dominant)	Overall	5	1.241	0.930–1.657	0.412	F	0.698	0
	European	2	1.003	0.587–1.715	0.990	F	0.715	0
	Asian	1	1.532	1.018–2.3305	0.0401	NA	NA	NA
TT vs. CC	Overall	3	2.396	0.682–8.423	0.173	F	0.306	15.5
	European	1	0.329	0.016–6.917	0.474	NA	NA	NA
	Asian	1	4.647	0.948–22.77	0.058	NA	NA	NA
Rs4986790 G vs. A allele	Overall	4	1.212	0.921–1.595	0.169	F	0.678	0
	European	1	0.861	0.459–1.614	0.640	NA	NA	NA
	Asian	1	1.355	0.935–1.965	0.109	NA	NA	NA
GG vs. GA + AA (Recessive)	Overall	3	2.416	0.552–10.58	0.242	F	0.259	25.9
	European	1	0.322	1015–6.773	0.466	NA	NA	NA
	Asian	1	7.161	0.854–60.01	0.070	NA	NA	NA
GG + GA vs. AA (Dominant)	Overall	4	1.188	0.882–1.601	0.257	F	0.860	0
	European	1	0.922	0.476–1.784	0.809	NA	NA	NA
	Asian	1	1.298	0.854–1.975	0.222	NA	NA	NA
GG vs. AA	Overall	3	2.482	0.566–10.88	0.228	F	0.247	28.3
	European	1	0.322	0.015–6.773	0.466	NA	NA	NA
	Asian	1	7.547	0.897–63.48	0.063	NA	NA	NA

F: Fixed effect model; R: Random effect model, NA: Not available.

### 3.3 The heterogeneity between studies and publication bias

Meta-analyses of TLR7 variations showed variations between the trials (Table 2, Table 3). However, ethnic meta-analyses showed decreased variability and revealed studies with similar impact values (Table 2, Table 3). The exclusion of studies with control genotypes that deviated from HWE had no effect on the results of the meta-analysis. It was difficult to establish a link between the funnel graphs, which are frequently used to identify publishing bias, owing to the small number of included studies. No meaningful findings were obtained from Egger's publishing bias regression test (P > 0.1).

## 4 Discussion

The meta-analysis revealed that the TLR7 rs179008 T variant was not associated with SLE. Furthermore, no causal relationship between TLR7 rs179008 T and SLE was found in individuals from Europe or Latin America, and the TLR4 variants rs4986791 and rs798690 did not increase the risk of SLE in Europeans. However, significant associations between SLE and the TLR7 rsrs3853839 GG + GA variant were found in both Arab and Asian groups. The TLR7 rs179010 T gene was also found to be associated with SLE in Asian populations. These findings suggest that genetic differences in TLR7 may play a role in the development of SLE in some groups. Considering the relationship between this polymorphism and SLE susceptibility in Arabs and Asians, it is possible that this association between TLR7 rs3853839 polymorphism and SLE risk is population-specific. The rs3853839 variation has been proposed to alter TLR7 function, leading to disruption of the immunological response and greater vulnerability to SLE. Similarly, the susceptibility of Asians to SLE was found to be associated with the TLR7 rs179010 T allele. The rs179010 T variant has been suggested to alter TLR7 expression or function, leading to immune system dysfunction and a higher susceptibility to SLE.

The discovery that TLR7 alteration is associated with a higher risk of SLE provides insight into the fundamental processes underlying this condition [31]. In the natural immune system, the TLR7 receptor plays a crucial role in the recognition of viral RNA and in triggering the production of type I interferons and other pro-inflammatory mediators [32]. TLR7 stimulation can produce autoantibodies against nuclear elements, such as DNA and RNA, which are indicative of SLE. Considering that women are more prone to acquiring SLE than men and that TLR7 is located on the X chromosome, the X chromosome may be crucial in the etiology of SLE [33]. Women have two copies of the X chromosome, and the loss of one copy can cause patchy expression of X chromosome genes. The greater susceptibility of females to SLE has been proposed to be caused by the amplification of TLR7 on the dormant X chromosome. In addition to its role in the innate immune response, TLR7 may contribute to the adaptive immune response [5]. The generation of autoantibodies against nuclear components is triggered by the stimulation of TLR7, which promotes B cell activation and division [34]. TLR7 activation can also enhance the presentation of autoantigens to T cells and promote the growth of autoreactive T cells. TLR7 stimulation leading to the production of autoantibodies is clinically relevant, considering the well-known specificity of anti-double-stranded DNA (anti-dsDNA) antibodies for SLE diagnosis. Despite being indicative of SLE, anti-dsDNA antibodies can also be present in autoimmune hepatitis (AIH), historically referred to as ‘lupoid hepatitis.’ Granito et al. stressed the diagnostic role of anti-dsDNA antibodies and highlight their presence in AIH and emphasized the need to consider AIH in patients with ANA and anti-dsDNA antibody positivity, underlining potential clinical and immunological similarities between SLE and AIH [2]. No association between SLE susceptibility and TLR7 rs179008, TLR4 rs4986791, or rs798690 SNPs was observed; however, further studies are required in this field owing to limited data.

This study has some limitations. First, the efficacy of the meta-analysis may have been hampered by the small number of included studies. Second, the included studies differed in terms of quality, with some having small sample sizes and the potential for publication bias. Third, the ethnic variation in the study groups was homogeneous. Therefore, further studies are needed to determine whether the TLR7 and TLR4 alleles are associated with SLE risk in more diverse groups. The strengths of this study include the use of numerous genetic models to evaluate the correlations between genetic variants and SLE risk and the substantial sample size [[35], [36], [37]], which provided adequate statistical power to identify meaningful relationships.

In conclusion, the results of the meta-analysis showed that TLR7 rs3853839 variant is associated with SLE risk in Asians and Arabs, and the TLR7 rs179010 T gene is associated with SLE in Asians. However, TLR7 rs179008, TLR4 rs4986791, and rs798690 SNPs are not associated with SLE risk. These findings suggest that genetic variables may contribute to the variability of the disorder and that the TLR7 pathway may be involved in the onset of SLE. The therapeutic relevance of TLR7 and TLR4 variants in the identification and management of SLE, as well as the fundamental processes underlying these associations, require further exploration.

## Funding sources

None.

## Data availability statement

Data included in article/supplementary material/referenced in article.

## Ethics statement

Review and/or approval by an ethics committee was not needed for this study because this research was a meta + -analysis.

## CRediT authorship contribution statement

**Young Ho Lee:** Writing – review & editing, Writing – original draft, Visualization, Validation, Supervision, Software, Resources, Project administration, Methodology, Investigation, Funding acquisition, Formal analysis, Data curation, Conceptualization. **Gwan Gyu Song:** Writing – review & editing, Validation, Methodology, Formal analysis, Conceptualization.

## Declaration of competing interest

The authors declare that they have no known competing financial interests or personal relationships that could have appeared to influence the work reported in this paper.
